# Ovary and fallopian tube as contents of indirect inguinal hernia: A case report

**DOI:** 10.1016/j.ijscr.2022.107733

**Published:** 2022-10-12

**Authors:** Shahzaib Maqbool, Ayesha Huma, Muhammad Idrees Anwar, Muhammad Atif Khan, Ka Yiu Lee

**Affiliations:** a Department of Surgery Unit II, Holy Family Hospital, Rawalpindi, Pakistan; b Department of Health Sciences, Mid Sweden University, Sweden

**Keywords:** Inguinal hernia, Ovarian hernia, Hernioplasty

## Abstract

**Introduction and importance:**

Ovarian and fallopian tube indirect inguinal hernias are rare hernias encountered on surgical floor. Herein, we are reporting a rare happening of obstructed ovarian and fallopian tube indirect inguinal hernia in an unmarried female.

**Presentation of case:**

A 19 years old unmarried female presented to surgical emergency with complain of swelling in left inguinal region that was about 3 × 3 cm on visual inspection from last 4 days that was associated with menstruation. Abdominal ultrasound (USG) was showing left ovarian and fallopian tube indirect inguinal hernia with preserved internal follicles and intact vasculature. The diagnosis of obstructed ovarian and fallopian tube indirect inguinal hernia was made and diagnostic laparoscopy and left sided hernioplasty was performed. Ovary was retrieved back into abdominal cavity and stitched to lateral pelvic wall.

**Discussion:**

Inguinal hernia itself is a rare happening in females and the presence of ovary and fallopian tube along with other hernial content can become a topic of discussion due to rarity of this case. It is of paramount significance to diagnose such cases with great expertise in order to avoid long term complications in terms of preserving fertility among females. Early utilization of radiological investigations like USG abdomen and laparoscopic retrieval of adnexal structures are standard approach in management of such cases.

**Conclusion:**

Presence of ovary and fallopian tube in indirect inguinal hernia is a rare happening that need to be diagnosed and treated at earliest to avoid infertility among females.

## Highlights

• Presence of ovary and fallopian tube as content of indirect inguinal hernia is a rare happening.
• Management of ovary and fallopian tube indirect inguinal hernia should be ascertained in timely manners.
• Colour doppler USG is of great significance in determining the presence of blood flow to ovary and fallopian tube.
• Laparoscopically diagnosis and retrieval of hernial contents is standard of care protocol.

## 1 Introduction

Inguinal hernia is a rare happening in females accounting for 5 % of the cases [1]. Though it's very less frequent in females, but it should be investigated in order to rule out hernia obstruction, strangulation (loss of blood supply). Inguinal hernia surgery is the most commonly Performed surgery on surgical floors. The symptoms severity is of variable spectrum ranging from asymptomatic to severe abdominal pain due to strangulation [2]. Incomplete closure of canal of nuck causes development of inguinal hernia in females [3].

The usual contents of inguinal hernia are small bowel along with omentum, however surgeons also come across some unusual contents like sigmoid colon, cecum, appendix, urinary bladder and uterus. The presence of ovary, fallopian tube and uterus as contents of inguinal hernia is a rare happening [4]. Herein we have reported a rare case of ovarian inguinal hernia in a reproductive age group female. This work has been reported in line with the SCARE 2020 criteria [5].

## 2 Presentation of case

**Fig. 1:**
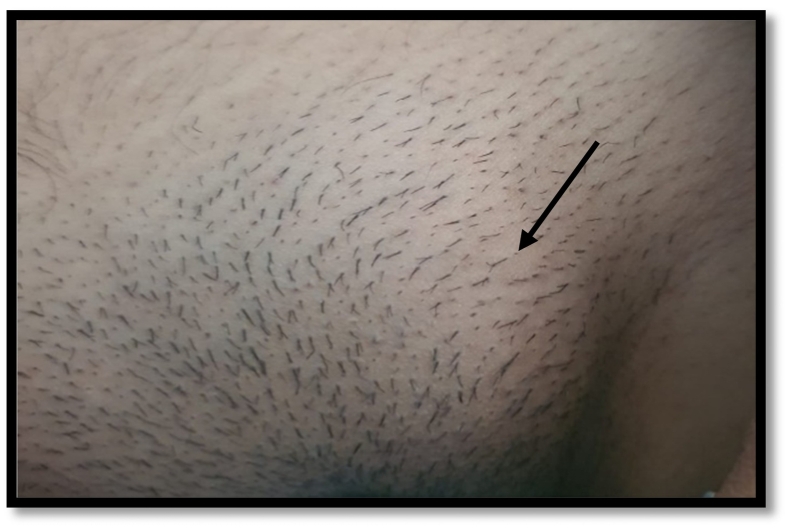
Showing swelling in left inguinal region.

A 19 years old unmarried female having no known comorbidities presented to surgical emergency with complain of swelling in left inguinal region that was about 3 × 3 cm on visual inspection from last 4 days. Patient also complaint of pain in the area of swelling that was gradual in onset, and progressive in nature and associated most of the time with menstruation and no other associated symptoms. The level of swelling also changes with menstruation. The patient was vitally stable otherwise. The swelling observed in left inguinal region is given in the (Fig. 1).

On examination patient was vitally stable except slight tachycardia (pulse rate:102/min). On examination of the inguinal region a 3 × 3 cm swelling was noted that was tender to touch, immobile, irreducible with absent cough impulse, however no skin and temperature changes were observed at the swelling site. The laboratory investigations were performed that were within reference values. Patients past medical and surgical history was insignificant. However, patient's gynaecological history was showing irregular menstruation from 3 to 5 days, however; the flow was regular.

**Fig. 2:**
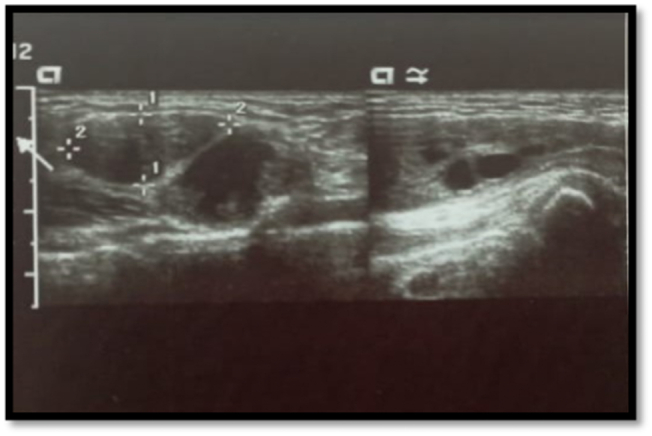
USG showing ovary in inguinal Canal.

Abdominal ultrasound (USG) was also performed that was showing left inguinal hernia containing left ovary with internal follicles and vascularity, and congenital agenesis of left kidney as shown in (Fig. 2). Similarly, USG inguinal region with complementary MR T2 cuts was also performed that was showing a well-defined sac filled with fluid forming mass like protrusion on superficial ring with ovary and fallopian tube in it. The sac measuring 7.2 × 4.9 cm was noted and ovary measuring 6.14 × 5.9 × 1.7 cm in size with internal follicles were observed. A complex haemorrhagic cyst measuring 2.8 × 1.6 × 2.2 cm was noted in left ovary. Uterus was tilted towards right side and right ovary was normal.

**Fig. 3:**
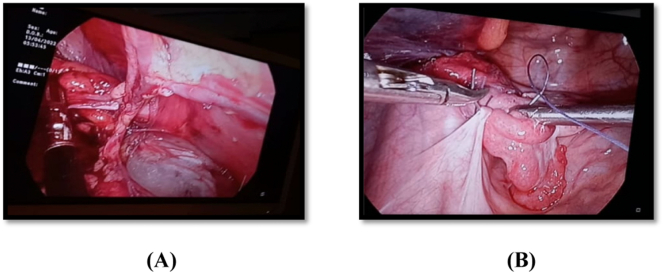
A&B. showing the ovary coming out of the inguinal canal (A), and being stitched with lateral pelvic wall (B).

The hernial sac containing gut loops and mesenteric fat were also seen on USG abdomen and pelvis. However, no other congenital or genetic abnormality was detected. The diagnosis of obstructed ovarian inguinal hernia was made and patient was prepared for surgery. After complete anaesthesia fitness patient was shifted to operation theatre and diagnostic laparoscopy and left sided hernioplasty was performed. Ovary was retrieved back into abdominal cavity as shown in (Fig. 3A) and stitched to lateral pelvic wall as shown in (Fig. 3B). Mesh was placed on hernial orifice and no complication was observed. The left ovary and fallopian tube were healthy when retrieved from inguinal canal. The unicornuate uterus and right sided ovary and fallopian tube were also healthy.

## 3 Discussion

Inguinal hernia is the defined as protrusion of abdominal viscera and preperitoneal fat into the inguinal canal. Inguinal hernias are the most frequently encountered benign surgical condition that accounted for about 75 % of the cases of abdominal hernias [6]. As far as gender wise distribution is concerned, the inguinal hernia most commonly affect male population accounting from 27 %–43 % of the cases as compared to female population who are less prone to inguinal hernias with their percentages varying from 3 to 6 % of the cases [7]. So, the prevalence of inguinal hernias, are extremely rare among females. The most commonly structured encountered in inguinal hernia are mostly peritoneum, colon, preperitoneal fat and its very rare to have the other contents like adnexa in females containing ovaries and fallopian tubes [8]. Most of the ovarian inguinal hernias are commonly observed in neonates and infants due to some associated congenital anomalies, but having ovarian inguinal hernia in a reproductive age group female is a rare happening and is of paramount significance [9]. In women of reproductive age, taking into account the rarity of this clinical condition, a high clinical suspicion is paramount to ensuring fertility and diagnosing potential simultaneous genital anomalies.

The diagnosis of inguinal hernias is based most commonly on clinical examination and sometimes patients sign and symptoms can also be the leading points towards the nature of diagnosis, as the patients with strangulated and obstructed inguinal hernias can be differentiated on the basis of nature and severity of pain at the mass region [10]. However, the Radiological investigations can also be used as an adjunct to diagnose ovarian inguinal hernia as the early utilization of Ultrasonographic evaluation along with colour Doppler ultrasound (CDUSG) can be of significant value in order to identify the nature of structure and blood supply of the herniated structures to differentiate it either obstructed or strangulated (ovarian torsion) [11]. In above mentioned case, the USG examination was showing the presence of left ovary along with fallopian tube in inguinal hernia, however, the blood supply ovary and fallopian tube was intact leading to the diagnosis of obstructed ovarian inguinal hernia. As far as the treatment of the ovarian inguinal hernia is concerned, the repositioning of the ovary of affected side along with fallopian tube and primary repair of the inguinal hernia is the best advised treatment option [2], but in our case along with repositioning and primary repair of left inguinal hernia the herniated structures like ovary and fallopian tube were also stitched with lateral pelvic wall to avoid future herniation.

The purpose of highlighting this particular case was not only the rare happening of ovarian inguinal hernia in females that accounted for only 3 %, but also to highlight the delicate nature of the case as far as female fertility and hernia related complications are concerned and most importantly, the association of genetically or congenitally triggered anomalies can also be the hick in such cases that need to be addressed.

## 4 Conclusion

It is a rare case of inguinal hernia in an unmarried female having left ovary and fallopian tube along with part of colon and mesentery as content of inguinal canal. This is a rare happening to have adnexal contents in inguinal canal accounting for only 3 % of the cases of inguinal hernias. These cases of ovarian inguinal hernias require special attention in order to avoid complications like ovarian torsion, strangulation and loss of fertility among females. In our case the diagnosis of obstructed ovarian inguinal hernia was made and involved ovary and fallopian tube were also preserved.

## Consent

Written informed consent was obtained from the patient for the publication of this case report and accompanying images. A copy of the written consent is available for review by the Editor-in-Chief of this journal on request.

## Funding

This work received no specific grant from any funding agency in the public, commercial, or not-for-profit sector.

## Ethical approval

Ethical approval has been exempted by our institution because this is a case report and no new technique were carried out.

## Registration of research studies

Not applicable.

## Guarantor

The Guarantor of this case report is Shahzaib Maqbool.

## Provenance and peer review

Not commissioned, externally peer-reviewed.

## CRediT authorship contribution statement

Shahzaib Maqbool: Writing-Original draft preparation & editing.

Ayesha Huma: Writing-review & editing.

Muhammad Atif Khan: Performed surgery & writing-review.

Muhammad Idrees Anwar, Ka Yiu Lee: review.

All authors read and approved the final manuscript.

## Declaration of competing interest

All the authors certify that there is no conflict of interest regarding the material discussed in the manuscript.
